# High cut-off dialysis mitigates pro-calcific effects of plasma on vascular progenitor cells

**DOI:** 10.1038/s41598-020-80016-7

**Published:** 2021-01-13

**Authors:** Theres Schaub, Daniel Janke, Daniel Zickler, Claudia Lange, Matthias Girndt, Ralf Schindler, Duska Dragun, Björn Hegner

**Affiliations:** 1 Clinic for Nephrology and Intensive Care Medicine, Charité – Universitätsmedizin Berlin, Corporate Member of Freie Universität Berlin, Humboldt-Universität Zu Berlin, and Berlin Institute of Health, Campus Virchow-Clinic, Augustenburger Platz 1, 13353 Berlin, Germany; 2 Institute of Cell Biology and Neurobiology, Charité – Universitätsmedizin Berlin, Corporate Member of Freie Universität Berlin, Humboldt-Universität Zu Berlin, and Berlin Institute of Health, Berlin, Germany; 3 Clinic for Stem Cell Transplantation, Department of Cell and Gene Therapy, University Medical Center Hamburg-Eppendorf, Hamburg, Germany; 4 Department of Internal Medicine II, Martin-Luther-University Halle-Wittenberg, Halle, Germany; 5 Berlin-Brandenburg School for Regenerative Therapies (BSRT), Berlin, Germany; 6 Center for Cardiovascular Research (CCR), Charité University Hospital, Berlin, Germany; 7 Vivantes Ida Wolff Hospital for Geriatric Medicine, Juchaczweg 21, 12351 Berlin, Germany

**Keywords:** Cardiovascular models, Cardiovascular diseases, Kidney diseases, Kidney, Kidney diseases, Renal replacement therapy, Cardiovascular biology, Mesenchymal stem cells

## Abstract

Mortality of patients with end-stage renal disease tremendously exceeds that of the general population due to excess cardiovascular morbidity. Large middle-sized molecules (LMM) including pro-inflammatory cytokines are major drivers of uremic cardiovascular toxicity and cannot be removed sufficiently by conventional high-flux (HFL) hemodialysis. We tested the ability of plasma from 19 hemodialysis patients participating in a trial comparing HFL with high cut-off (HCO) membranes facilitating removal of LMM to induce calcification in mesenchymal stromal cells (MSC) functioning as vascular progenitors. HCO dialysis favorably changed plasma composition resulting in reduced pro-calcific activity. LMM were removed more effectively by HCO dialysis including FGF23, a typical LMM we found to promote osteoblastic differentiation of MSC. Protein-bound uremic retention solutes with known cardiovascular toxicity but not LMM inhibited proliferation of MSC without direct toxicity in screening experiments. We could not attribute the effect of HCO dialysis on MSC calcification to distinct mediators. However, we found evidence of sustained reduced inflammation that might parallel other anti-calcifying mechanisms such as altered generation of extracellular vesicles. Our findings imply protection of MSC from dysfunctional differentiation by novel dialysis techniques targeted at removal of LMM. HCO dialysis might preserve their physiologic role in vascular regeneration and improve outcomes in dialysis patients.

## Introduction

Despite advances over the last decades, morbidity and mortality in patients with end-stage renal disease (ESRD) especially due to cardiovascular complications remain unacceptably high^1^. A plethora of life-style interventions and pharmacologic therapies targeting traditional cardiovascular risk factors such as hypertension, diabetes and hypercholesterolemia as well as nontraditional risk factors including disturbed calcium-phosphate-parathyroid hormone metabolism have been studied but only few of them have proven beneficial^2^. Thus, the uremic syndrome, caused by about 150 known and probably many more unknown uremic retention solutes (URS) with a large variety of toxic properties^3^, remains an unsolved medical problem. While current dialysis procedures effectively remove small water soluble molecules up to 15 kDa, members of two other classes of URS, large middle molecules (LMM) with a molecular weight > 15 kDa and protein bound molecules (PBM), are difficult to be removed. Yet, many LMM and PBM including pro-inflammatory cytokines, parathyroid hormone, p-cresyl sulfate and indoxyl sulfate have been identified as major drivers of pathologies associated with the uremic syndrome^3^. Hemodiafiltration (HDF) had been developed to remove LMM more efficiently but there is increasing evidence that HDF does not provide a survival advantage over conventional high-flux (HFL) hemodialysis independently of convective volumes in clinical practice^4^. An alternative promising strategy with recent technical advances is enhanced removal of LMM by dialysis membranes with higher molecular weight cut-offs (HCO) between 50 and 60 kDa similar to healthy kidneys^5^. As an example, this approach has been shown to reduce systemic inflammation in maintenance hemodialysis patients^6^, but effects on cardiovascular pathologies and clinical end-points have not been studied in detail.

Severe calcification of the tunica intima and tunica media of arteries, the hallmark of uremic vasculopathy, is a key determinant of cardiovascular risk in ESRD^7^. Vascular regeneration depends on local vascular precursor cells, the pericytes^8,9^, that can differentiate into mature vascular cells such as vascular smooth muscle cells (VSMC) and are replenished by mesenchymal stromal cells (MSC)^10^. MSC are progenitor cells residing in a perivascular niche^11^ that can be isolated from all vascularized tissues^12^. They feature multi-lineage differentiation potential including VSMC^13^ and osteoblast phenotypes in combination with high regenerative capacity also for the vasculature^14^. Active transformation of VSMC to calcifying cells with an osteoblast-like phenotype similar to intramembranous and enchondral bone formation is a key process in arterial calcification^7,15^. Yet, a majority of osteoblast-like cells in calcifying intimal and medial vascular lesions forming in a mouse model of chronic kidney disease have been found to be derived from MSC-like cells in the arterial adventitia^16^. Thus, protection of the undifferentiated physiologic state of MSC preserving their regenerative properties holds promise to offset accelerated vascular calcification in patients suffering from chronic kidney disease more effectively than strategies solely targeted at VSMC with a terminally differentiated phenotype.

In a previous study, we identified the three LMM interleukin-1β (IL-1β), tumor necrosis factor-α (TNF-α), and fibroblast growth factor-2 (FGF-2) as the strongest inducers of osteoblastic transformation of MSC in an unbiased screening approach testing 63 individual URS^17^. Pharmacologic blockade of IL-1β, TNF-α and FGF in combination potently protected MSC from calcifying phenotype conversion^17^. However, clinical application of small-molecules or biological agents targeting multiple pro-inflammatory cytokines and FGF-2 can be costly and associated with considerable side effects. Enhanced removal of LMM by HCO dialysis holds promise to preserve MSC regenerative capacity without excessive costs and risks. In this report, we describe the effects of HCO dialysis on osteoblastic differentiation and calcification of MSC and investigate possible protective mechanisms.

## Results

### Dialysis with HCO membranes reduces the ability of patient plasma to induce osteoblastic differentiation in MSC

**Figure 1:**
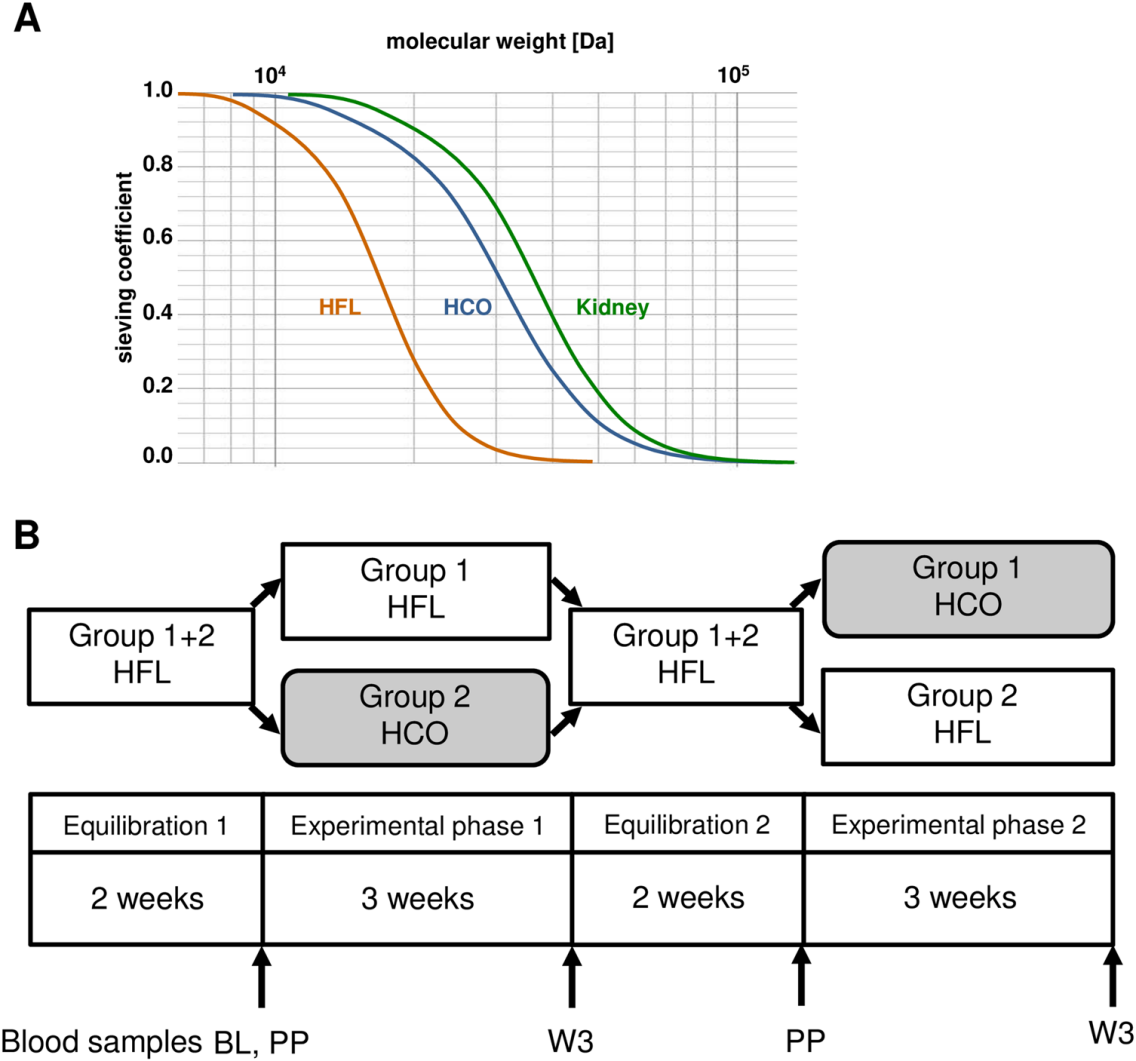
Clinical trial comparing HFL with HCO dialysis membranes. (**A**) Scheme showing the elimination characteristics of HFL and HCO membranes in comparison to healthy kidneys with sieving coefficient plotted against molecular weight. (**B**) Cross over-design of the randomized study with 2 cycles of equilibration and experimental phases. BL: Baseline blood samples for clinical chemistry drawn pre-dialysis at the first dialysis session of experimental phase 1. PP: Experimental blood samples drawn pre- and post-dialysis at the first dialysis session of experimental phases 1 and 2. W3: Experimental blood samples drawn pre-dialysis at the first dialysis session after completion of the 3-week experimental phases 1 and 2.

We had access to sera from a subgroup of 19 patients participating in a randomized controlled clinical trial testing conventional HFL dialyzers against dialyzers characterized by a higher molecular weight cut-off (HCO; Fig. 1A) in maintenance hemodialysis^6^. In a cross over-design as shown in Fig. 1B, stable dialysis patients were first treated with an HFL membrane for 2 weeks (equilibration 1) and were then divided into 2 groups: One group was continued on the HFL membrane while the other group was switched to an HCO membrane for 3 weeks (experimental phase 1). After a wash out-phase of 2 weeks where all patients were dialyzed with the HFL membrane (equilibration 2), patients that were treated with the HFL membrane in experimental phase 1 received the HCO membrane in experimental phase 2 whereas patients treated with the HCO membrane in experimental phase 1 were now continued on the HFL dialyzer for another 3 weeks (experimental phase 2). To specifically study the effects of these different dialysis modalities on osteoblastic transformation and calcification of vascular progenitors, we took advantage of an in vitro model using human MSC isolated from bone marrow aspirates of 20 healthy donors as described earlier^17^. Pre-dialysis plasma after 3 weeks of dialysis with either an HFL membrane or with an HCO membrane was tested.

**Figure 2:**
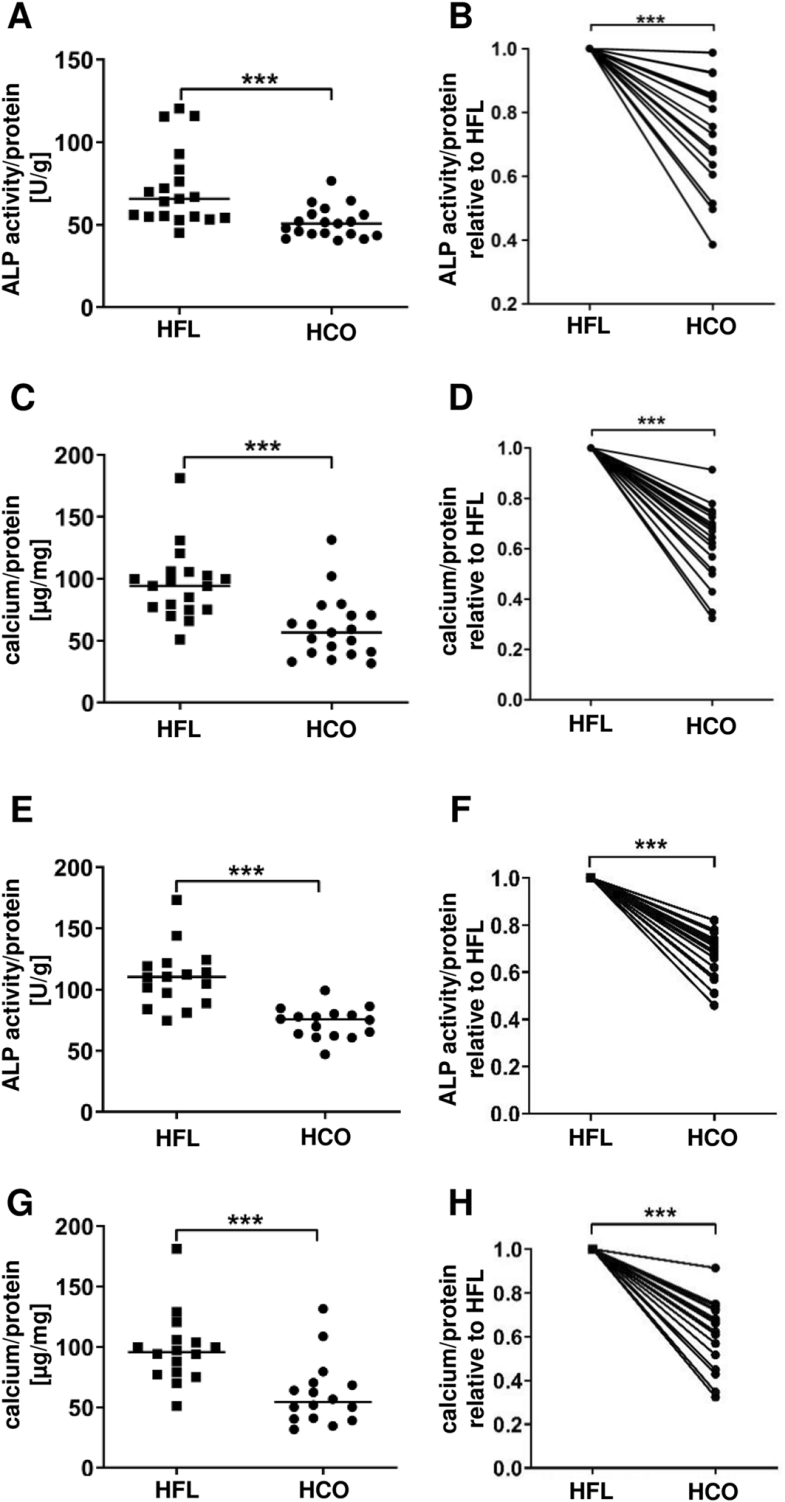
Effect of dialysis with HCO membranes on plasma induced osteoblast differentiation and calcification of MSC. (**A**–**D**) MSC were incubated with OM containing plasma from dialysis patients treated either with conventional HFL dialyzers or from the same patients after a 3-week course of dialysis with HCO membranes. n = 19. (**A**,**B**) ALP activity and (**C**,**D**) calcium deposition normalized to protein content measured on single-patient level. (**B**) ALP activity and (**D**) calcium deposition with HCO plasma relative to HFL plasma (set to 1.0) showing reduced osteoblastic differentiation and calcium deposition for every single patient. (**E**–**H**) Plasma from all 19 patients were combined to 4 different plasma pools for both treatment modalities. Each plasma pool was applied to 4 MSC preparations from different healthy donors. n = 16. (**E**,**F**) ALP activity and (**G**,**H**) calcium deposition were normalized to protein content. (**F**) ALP activity and (**H**) calcium deposition with HCO plasma relative to HFL plasma (set to 1.0) showing reduced osteoblastic differentiation and calcium deposition for every single combination of a plasma pool and MSC preparation. ALP activity was assessed after incubation for 7 days. Calcium deposition was measured after incubation for 3 weeks. ***P < 0.001.

Overall, the potential for induction of osteoblastic differentiation in MSC was reduced in plasma obtained under HCO dialysis compared to HFL dialysis as indicated by ALP activity (51.52 ± 2.2 U/g protein versus 72.08 ± 5.3 U/g protein; Fig. 2A). Paired analysis of the two samples from each patient revealed that ALP activity was lower after exposure to HCO-plasma compared to HFL-plasma for every individual patient (Fig. 2B). Reduction rates varied between 0.02 and 0.61 (Fig. 2B). Calcium deposition was also decreased when MSC were exposed to plasma from patients treated with HCO membranes compared to plasma obtained during a period of HFL dialysis (60.2 ± 5.8 µg/mg protein versus 95.4 ± 6.5 µg/mg protein; Fig. 2C). Similar to ALP activity, this result was found for every single patient with reduction rates in a range from 0.09 to 0.68 (Fig. 2D). To exclude the possibility that the observed effects depended on the MSC preparation used in the experiments, we combined the plasmas from all 19 patients to 4 different plasma pools that were all applied to MSC preparations from 4 different donors. Again, ALP activity and calcium deposition were lower when cells were exposed to HCO-plasma pools in comparison to HFL-plasma pools independently of the MSC preparation (Fig. 2E,G). The same effect was seen for every single combination (Fig. 2F,H).

### HCO dialysis reduces mediators with a molecular weight between 24 and 66 kDa more effectively than HFL dialysis

**Figure 3:**
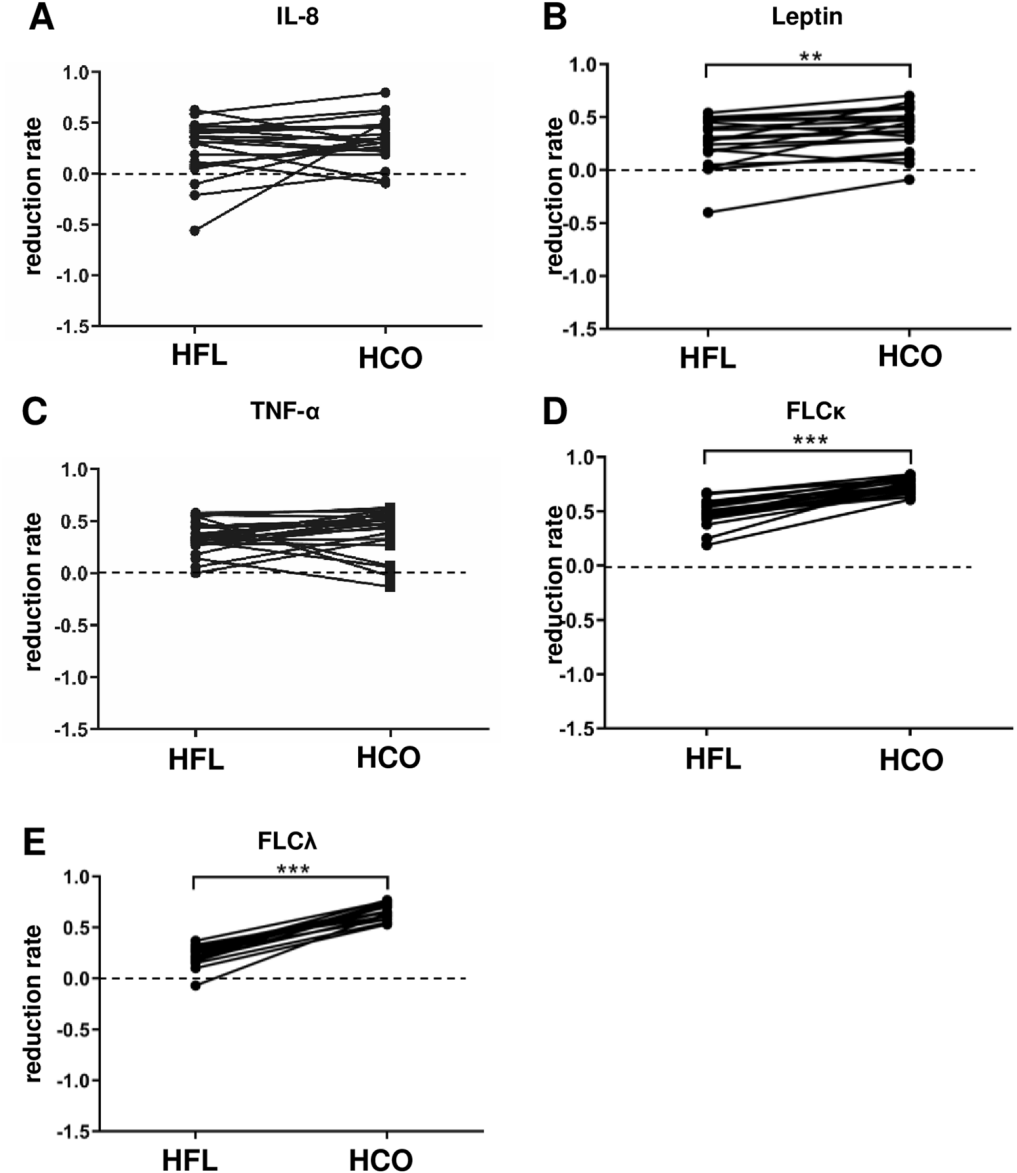
Mediators that were reduced after both a single HFL and HCO dialysis. Blood samples were collected pre- and post-dialysis at the first dialysis session of both experimental phases. Reduction rates were calculated as 1-(concentration post-dialysis/concentration pre-dialysis). (**A**) IL-8, n = 19; (**B**) Leptin, n = 19; (**C**) TNF-α, n = 19; (**D**) FLCκ, n = 19; (**E**) FLCλ, n = 19. **P < 0.01, ***P < 0.001.

**Figure 4:**
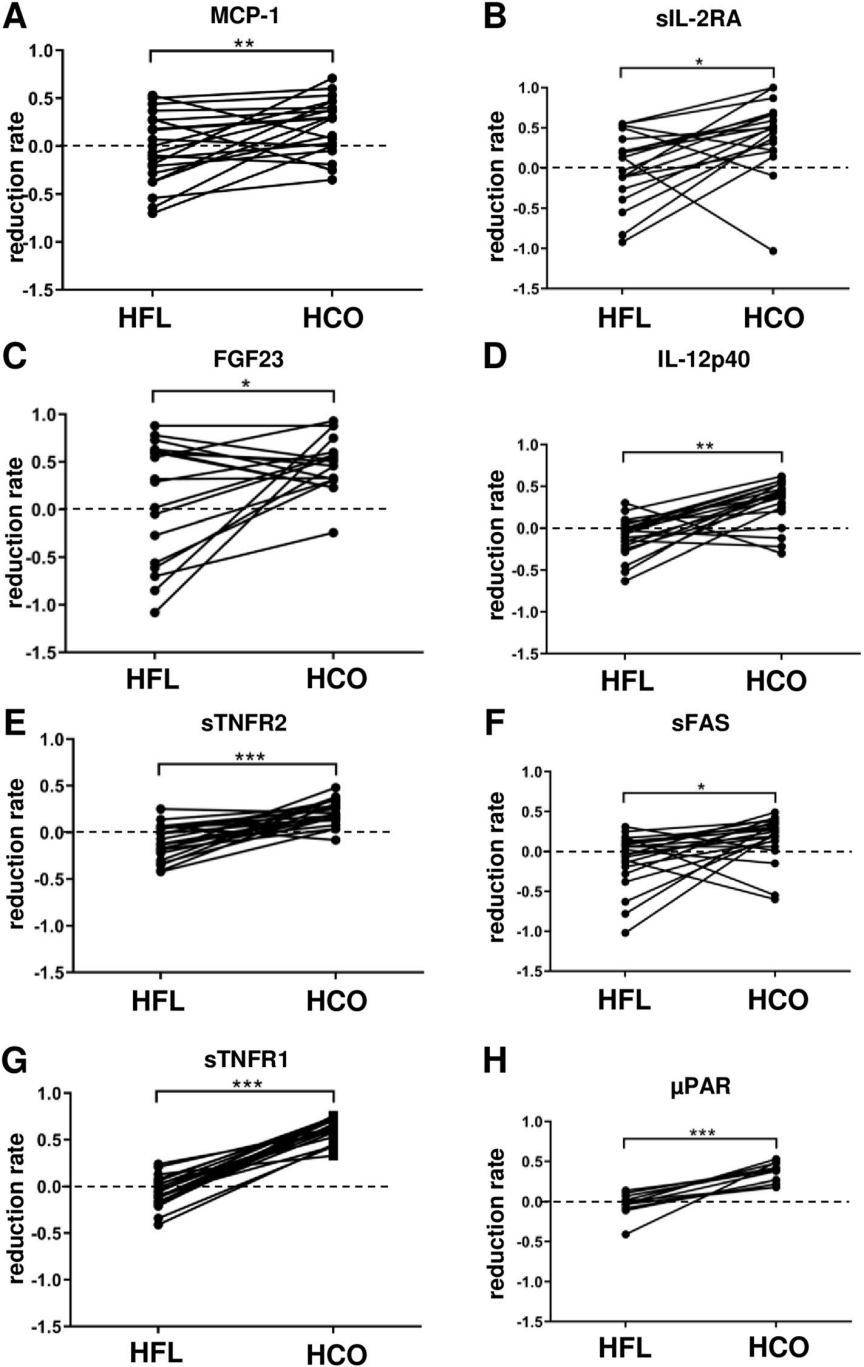
Mediators that were reduced after a single HCO but not HFL dialysis. Blood samples were collected pre- and post-dialysis at the first dialysis session of both experimental phases. Reduction rates were calculated as 1-(concentration post-dialysis/concentration pre-dialysis). (**A**) MCP-1, n = 19; (**B**) sIL-2RA, n = 19; (**C**) FGF23, n = 18; (**D**) IL-12p40, n = 19; (**E**) sTNFR2, n = 19; (**F**) sFAS, n = 19; (**G**) sTNFR1, n = 19; (**H**) µPAR, n = 11. *P < 0.05, **P < 0.01, ***P < 0.001.

An in-depth re-analysis of a broad panel of 33 mediators with potential impact on vascular calcification in chronic kidney disease (CKD) from the original study^6^ together with eight additional molecules covering a molecular weight range from 2.7 to 2500 kDa was performed on the plasma of the patients included in this sub-study to reveal potential mechanisms of reduced MSC osteoblastic differentiation. Standard clinical chemistry differed only with respect to albumin (66 kDa) which was lower in plasma obtained after 3 weeks of HCO dialysis than at baseline or after 3 weeks of HFL dialysis (Table 1). Several mediators with a molecular weight up to 22.5 kDa were reduced after a single dialysis with both HFL or HCO membranes (IL-8, leptin, TNF-α, free light chain kappa (FLCκ); Table 2, Fig. 3). However, reduction rates for leptin and FLCκ were significantly higher with HCO dialysis (Fig. 3B,D). MCP-1 was decreased only after treatment with HCO membranes (Table 2, Fig. 4A). In the segment between 24.5 and 61 kDa, only free light chain lambda (FLCλ) was significantly reduced by HFL dialysis while a single HCO dialysis resulted in lower levels of 8 mediators including sIL-2RA, FGF23, IL-12p40, sTNFR2, sFAS, FLCλ, sTNFR1, and µPAR (Table 2). Reduction rates for all 8 molecules were higher with HCO than with HFL membranes (Figs. 3E, 4B–H). Importantly, concentrations of sIL-2RA, FLCλ, and sTNFR1 were significantly lower in plasma obtained pre-dialysis after 3 weeks of treatment with HCO membranes than after 3 weeks with HFL membranes suggesting a long-lasting effect (Table 2). These results indicate superior removal of molecules accumulating in CKD including pro-inflammatory cytokines by HCO dialysis especially in the range of 24.5 to 66 kDa corresponding to LMM.

**Table 1:** Clinical chemistry at baseline and after 3 weeks of HFL and HCO dialysis.

		Reference	Baseline	HFL	HCO	P Kruskal–Wallis
Sodium	mmol/L	135–145	139 (137; 140)	138 (136; 139)	138 (137; 140)	0.494
Potassium	mmol/L	3.6–4.8	5.6 (4.6; 6.2)	5.5 (4.8; 6.2)	5.7 (4.6; 6.0)	0.962
Calcium	mmol/L	2.20–2.60	2.29 (2.19; 2.37)	2.25 (2.14; 2.39)	2.13 (2.03; 2.27)	0.077
Phosphate	mmol/L	0.8–1.5	1.5 (1.2; 1.8)	1.7 (1.4; 1.9)	1.8 (1.4; 2.0)	0.392
Creatinine	µmol/L	44–106	675 (562; 871)	688 (575; 851)	682 (605; 860)	0.986
Urea	mmol/L	1.7–9.3	19.4 (16.0; 22.1)	19.4 (13.7; 22.7)	15.8 (13.1; 20.2)	0.087
CRP	mg/L	< 5	9.2 (4.6; 10.1)	6.7 (4.5; 16.8)	6.0 (4.1; 10.0)	0.047
Albumin	g/L	38–54	35 (33; 37)	36 (33; 37)	30*** (26; 31)	< 0.001

Blood samples were collected pre-dialysis at the first dialysis session of experimental phase 1 (baseline) and pre-dialysis at the first dialysis session after completion of both 3-week experimental phases (HFL, HCO). Median (25% percentile; 75% percentile), n = 19, ***P < 0.001 HCO versus Baseline and HCO versus HFL.

**Table 2:**
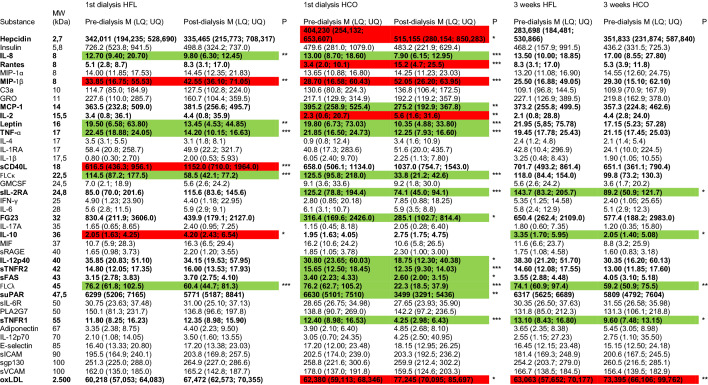
Plasma concentrations of a panel of 41 mediators at the beginning and the end of each experimental phase.

Blood samples were collected pre- and post-dialysis at the first dialysis session of each experimental phase and pre-dialysis at the first dialysis session after completion of the 3-week experimental phases. Green marks: lower concentrations post- versus pre-dialysis or after 3 weeks of HCO versus HFL dialysis. Red marks: higher concentrations post- versus pre-dialysis or after 3 weeks of HCO versus HFL dialysis. Median (25% percentile; 75% percentile), n ≤ 19, *P < 0.05, **P < 0.01, ***P < 0.001.

**Figure 5:**
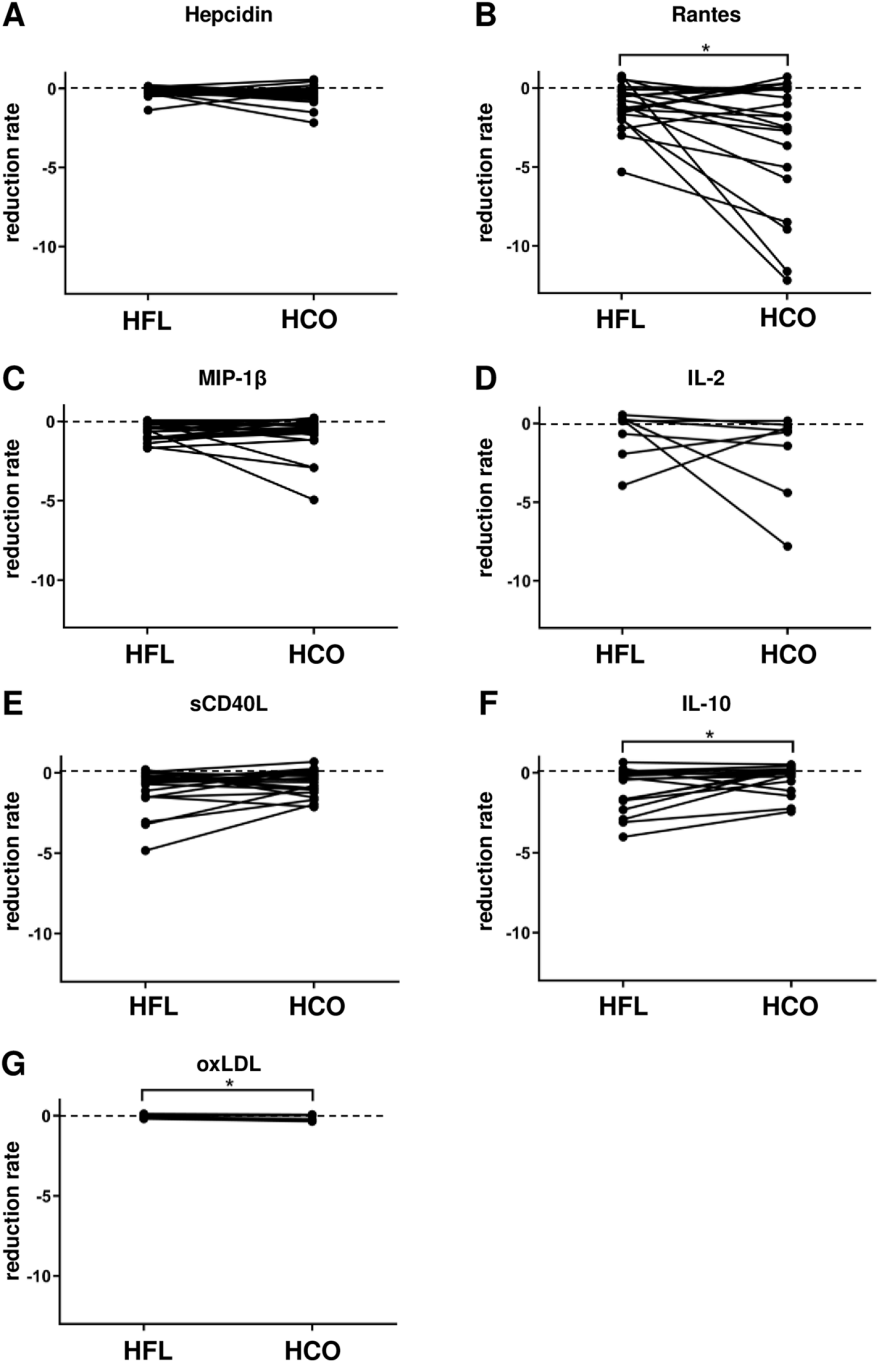
Mediators that were increased after either HFL or HCO dialysis or both. Blood samples were collected pre- and post-dialysis at the first dialysis session of both experimental phases. Reduction rates were calculated as 1-(concentration post-dialysis/concentration pre-dialysis). (**A**) Hepcidin, n = 19; (**B**) RANTES, n = 19; (**C**) MIP1b, n = 19; (**D**) IL-2, n = 8; (**E**) sCD40L n = 19; (**F**) IL-10, n = 17; (**G**) oxLDL, n = 9. *P < 0.05.

On the other hand, the concentrations of some mediators particularly in the low molecular weight range were increased after either HFL or HCO dialysis or both (Table 2) reflected by negative reduction rates (Fig. 5). Most of these mediators were pro-inflammatory cytokines (Rantes, MIP-1β, IL-2) or related to a pro-inflammatory state (hepcidin, sCD40L, oxLDL). IL-10 was the only anti-inflammatory mediator in this group and showed higher levels after a single HFL dialysis as well as after 3 weeks of HFL dialysis (Table 2) since the reduction rate was lower in the negative range compared to HCO dialysis (Fig. 5A). The only mediator with higher concentrations after 3 weeks of HCO dialysis was oxLDL (Table 2).

### FGF23 is a LMM capable of inducing osteoblastic differentiation in MSC

**Figure 6:**
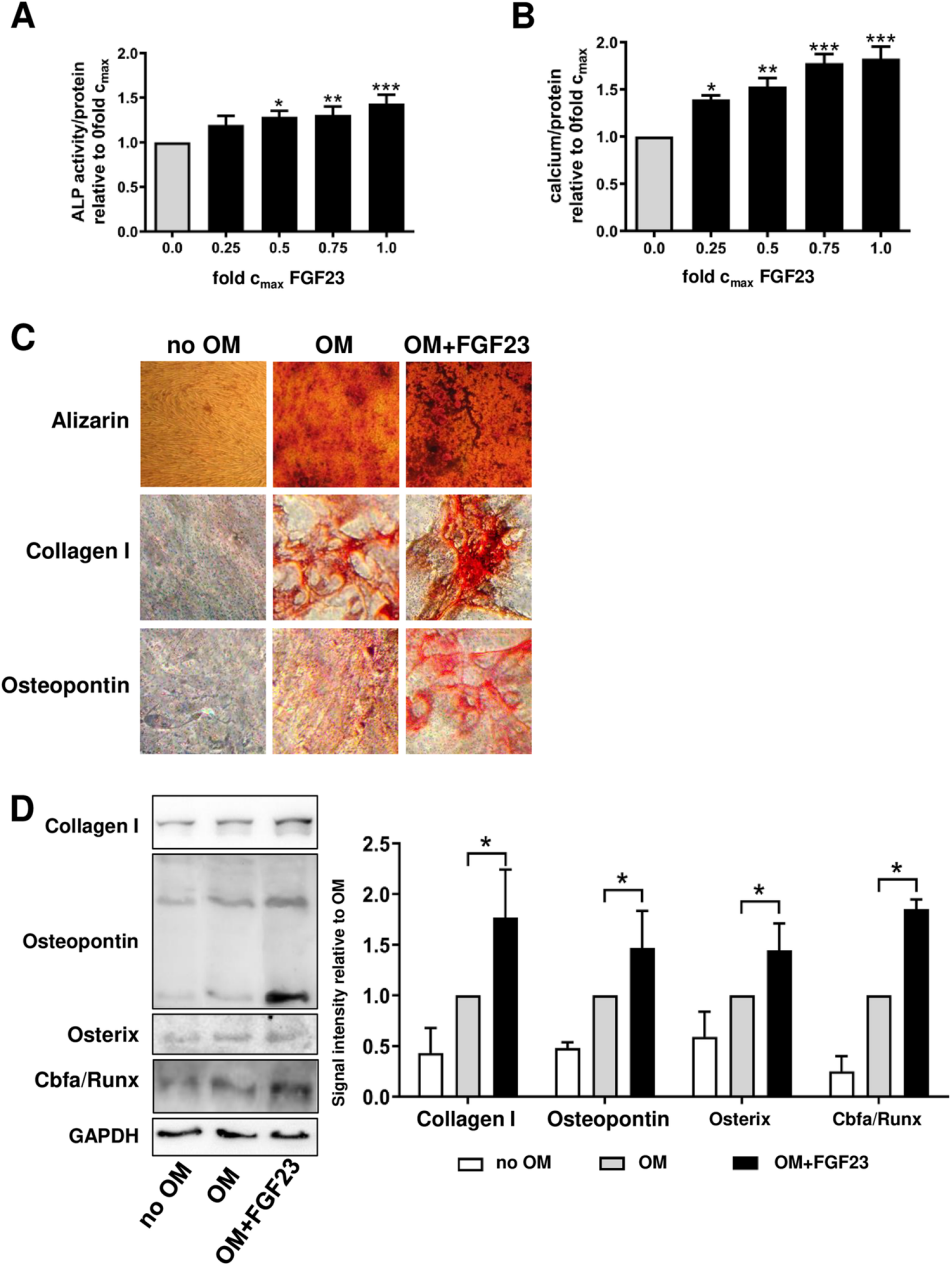
FGF23 induces osteoblastic differentiation in MSC. (**A**) ALP activity in MSC treated with different concentrations of FGF23 in OM for 7 days. (**B**) Calcium deposited by MSC cultured for 3 weeks with increasing concentrations of FGF23 in OM. “fold c_max_” denotes the x-fold concentration of the highest reported concentration found in uremic patients. Values were normalized to protein content and are expressed relative to OM without FGF23 (set to 1.00); n = 4. (**C**) Alizarin staining and immunocytochemistry for osteoblast marker proteins after incubation of MSC for 3 weeks in OM with FGF23 at the highest reported concentration in uremia. A representative experiment is shown. (**D**) Western blot analysis for expression of osteoblast marker proteins after treatment of MSC with OM and FGF23 at the highest concentration found in ESRD for 3 weeks. Representative blots and statistical analysis of 3 independent experiments are displayed. *P < 0.05, **P < 0.01, ***P < 0.001.

We asked, whether or not FGF23, a prototypical mediator of the LMM group characterized by a higher reduction rate with HCO compared to HFL membranes and significant removal by HCO dialysis, exerted pro-calcifying effects on MSC similar to IL-1β and TNF-α that had been identified as potent promotors of MSC calcification^17^. MSC have been shown to express FGF receptors 1–4 that signal upon binding of FGF23^18–20^. Incubation of MSC with FGF23 resulted in a dose-dependent increase in ALP activity (Fig. 6A) and deposition of calcified extracellular matrix (Fig. 6B,C). FGF23 treated cells stained positive for collagen I and osteopontin (Fig. 6C). These osteoblast marker proteins as well as Osterix and Cbfa/Runx were markedly upregulated as demonstrated by western blot analysis (Fig. 6D) indicating osteoblastic differentiation of MSC enhanced by FGF23.

### Screening of individual URS for proliferation and toxicity

**Figure 7:**
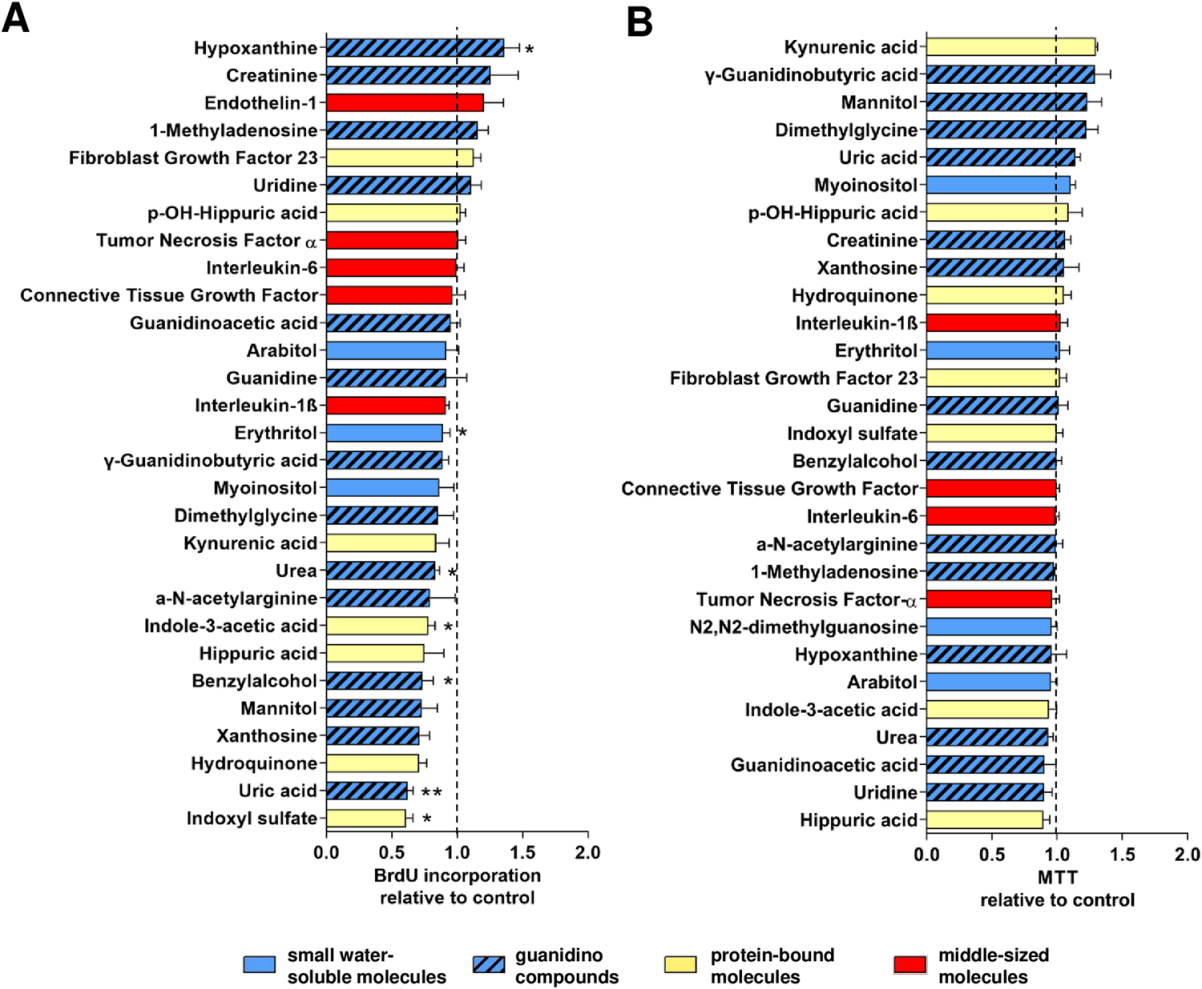
Screening of individual URS for proliferation and toxicity. (**A**) BrdU incorporation and (**B**) metabolism of MTT were measured after exposure of MSC to URS at the highest reported uremic concentrations for 24 h. Results are expressed relative to the appropriate solvent control (set to 1.00). Means + SEM, n ≤ 14, *P < 0.05, **P < 0.01.

Since vascular calcification is not only driven by osteoblastic differentiation of vascular cells and their progenitors but also by other mechanisms such as proliferation and cell death^21^ we extended our screening approach for biologic effects of URS on MSC. Proliferation of MSC in response to 29 individual URS at their highest reported concentration found in patients with ESRD^3,22–27^ was tested by measuring BrdU incorporation. Hypoxanthine increased proliferation by 36% (Fig. 7A) whereas erythritol, urea, benzyl alcohol, and uric acid, four other water soluble URS, reduced proliferation by 11–38% (Fig. 7A). The protein bound URS indoxyl sulfate and indole-3-acetic acid diminished proliferation by 39% and 22%, respectively (Fig. 7A). The MTT test was employed to test for reduced mitochondrial metabolic activity of MSC as a surrogate for toxicity. No acute cytotoxic effect was detected for any of the URS (Fig. 7B).

## Discussion

Restoration of endogenous vascular repair mechanisms in patients with ESRD appeals as a novel strategy to sustainably reduce cardiovascular disease burden and mortality in this highest-risk population. We studied the effect of HCO dialysis on phenotype conversion of MSC functioning as vascular progenitors to osteoblast-like calcifying cells involved in uremic media calcification. Pre-dialytic plasma obtained from patients after 3 weeks of HCO dialysis was more than one third less potent to facilitate osteoblastic differentiation and calcification in MSC compared to HFL dialysis. Several mediators with a molecular weight of 24–66 kDa were removed at a higher rate by HCO than by HFL dialysis including the prototypic LMM FGF23. We found that FGF23 had the ability to promote osteoblastic differentiation in MSC resulting in excess deposition of calcified extracellular matrix. Screening experiments of URS revealed that some water soluble or protein bound substances but no LMM impacted on proliferation of MSC without any signs of acute cytotoxicity. Further studies are needed to fully elucidate how HCO dialysis favorably modifies plasma composition to protect vascular progenitors from calcification.

The impressive reduction of calcium deposition by MSC undergoing osteoblastic differentiation described in this study adds to the growing body of evidence that advanced dialysis techniques aimed at better removal of LMM might reduce vascular calcification the perspective of preserved vascular regenerative capacity. Similar protective effects of dialysis with HCO membranes with elimination characteristics closer to those of healthy kidneys (Fig. 1A) have been described for the trans-differentiation of VSMC to osteoblast-like cells in vitro. Differentiated VSMC maintained their contractile phenotype decisive for physiologic vascular function^28^. Importantly, we now demonstrated that—after exposure to uremic plasma modified by HCO dialysis—VSMC progenitor cells from the pericyte-MSC continuum were resistant to osteoblastic differentiation and calcification. However, if the cells retain full ‘stemness’ with multi-lineage differentiation capacity and other features of undifferentiated MSC remains to be shown. Preserving the functionality of uncommitted precursors involved in vessel formation and vascular repair under physiologic conditions^29^ might be even more crucial to improve cardiovascular outcomes in dialysis patients since various entities of vascular pathologies such as media calcification and neointima formation develop from maladaptively differentiated vascular precursor cells^16,29^. In addition, paracrine effects exerted by locally resident or circulating bone marrow-derived stem cells have been implicated in angiogenesis and vascular repair^29,30^. Mechanisms include stabilization and repair of the endothelial layer as well as recruitment of circulating cells to sites of angiogenesis and vascular damage.

Our detailed analysis of 41 different molecules with a widely spread molecular weight range between 2.7 and 2500 kDa clearly demonstrated superior removal of plasma compounds belonging to the class of LMM between 24.8 and 66 kDa by HCO dialysis in comparison to HFL dialysis. We chose to exemplarily examine FGF23, a 32 kDa protein involved in phosphate and vitamin D metabolism, in more detail since it is strongly associated with all-cause and cardiovascular mortality throughout all stages of CKD^31^. While its role as a cardiac toxin causing myocardial remodeling with left ventricular hypertrophy and fibrosis is well-established, the involvement of FGF23 in uremic vascular calcification remains controversial^31^. Although higher levels of FGF23 positively correlate with aortic calcification in clinical studies^32,33^, it did not aggravate calcification of VSMC or aortic rings in vitro^34^. In CKD as a Klotho-deficient state, FGF23 appears to be even protective against VSMC calcification^31^. On the contrary, we found a clear dose-dependent amplification of osteoblastic differentiation and calcification of MSC by FGF23 (Fig. 6). A plausible mechanism is an FGF23-mediated induction of a senescent state as described by Sato et al.^20^ since cellular senescence is a hallmark of transition to a calcifying phenotype in MSC^35^. Thus, FGF23 might contribute to vascular calcification through negative effects on vascular progenitors rather than differentiated VSMC. Furthermore, FGF23 appears to form a pro-inflammatory vicious cycle together with IL-1β and TNF-α^31^, cytokines well-known for their potent calcifying effect on both VSMC^31^ and MSC^17^. Hence, indirect effects of FGF23 via pro-inflammatory cytokines might additionally be at play in uremic vascular calcification.

Nevertheless, in the pre-dialytic samples that were used to assess the influence of membrane permeability on osteoblastic differentiation of MSC besides albumin only four mediators, three of them considered anti-inflammatory, were detected at lower levels after 3 weeks of HCO dialysis compared to HFL dialysis: sIL-2RA, IL-10, FLCλ, and sTNFR1. In our previous studies, IL-10 at uremic concentrations did not enhance calcification of MSC^17^ and neither sIL-2RA nor sTNFR1 had an impact on calcification of VSMC over a broad range of concentrations^28^. From a different perspective, those URS with the highest potential for induction of osteoblastic differentiation in MSC as identified in an unbiased screening, IL-1β and TNF-α^17^, did not differ significantly between the two groups pre-dialysis after three weeks although there was a trend to lower IL-1β levels with HCO membranes. We chose to examine pre-dialytic instead of post-dialytic plasma because the concentrations pre-dialysis would reflect more reliably the composition of the extracellular milieu throughout the whole interdialytic interval. Based on these findings, we were not able to identify any LMM that could directly account for the observed protective effects of HCO dialysis on MSC calcification.

Since many protein-bound molecules including indoxyl sulfate and indole-3-acetic acid have been implicated in CKD-associated vascular pathologies^36,37^ it has been speculated that albumin loss caused by HCO dialysis could result in enhanced clearance of protein-bound URS with consecutively reduced propensity of plasma to induce calcification. However, protein-bound toxins did not influence osteoblastic differentiation of MSC in vitro as assessed by ALP activity after 7 days^17^. Moreover, dialysis with a medium cut-off (MCO) membrane designed to promote removal of LMM while retaining albumin^38^ for 12 weeks markedly reduced the ability of plasma to induce calcification of VSMC in vitro in comparison to HFL dialysis^39^ although differences in albumin levels were marginal^40^. These findings argue against a pivotal role for albumin-bound URS to explain the protective effect of HCO dialysis with regard to MSC calcification as described in this paper. On the other hand, we found an inhibitory effect of indoxyl sulfate and indole-3-acetic acid on proliferation of MSC (Fig. 7B) that could translate into impaired vascular regenerative properties contributing to the well-established adverse cardiovascular outcome associated with these URS.

From the list of differentially concentrated mediators after the 3-week experimental phases sTNFR1 stands out since higher levels have been shown to predict kidney disease progression, cardiovascular events and death in multiple CKD and non-CKD populations^41,42^. Although sTNFR1, a functional antagonist of TNF-α, might not exert toxic effects on the cardiovascular system itself, it might reflect a pro-inflammatory high-risk state as shedding of sTNFR1 is a key component of the inflammatory response^43,44^. Thus, lower levels of sTNFR1 might indicate a sustained reduction of inflammation by HCO dialysis while pro-inflammatory molecules themselves have already equilibrated at the end of the interdialytic interval. In the same line of evidence, transcripts of numerous pro-inflammatory mediators were downregulated in peripheral blood leukocytes pre-dialysis when MCO membranes were used^6^. Interestingly, sTNFR1 has also been attributed a role as a stabilizer of TNF-α augmenting some of its effects^45^. Consequently, reductions in sTNFR1 might not necessarily result in increased TNF-α activity, but might as well have an opposite, anti-inflammatory effect.

A potential powerful means of cardiovascular protection conferred by HCO dialysis not covered by our test system could be a favorable impact on extracellular vesicles (EV) such as exosomes and microparticles (MP): Circulating levels of MP derived from endothelial cells independently predict all-cause and cardiovascular mortality in hemodialysis patients^46^. CKD patients with vascular calcification have a higher burden of endothelial MP than those without vascular calcification and their endothelial MP were capable of inducing the osteoblast marker protein osteocalcin in VSMC in vitro^47^. As a link to inflammation, TNF-α stimulates the production of bone morphogenic protein-2 enriched endothelial MP that can promote transition from a contractile to a calcifying phenotype in VSMC^48^. Given a size between 30 and 1000 nm for EV^49^ and a pore size of 8–12 nm for the HCO membrane^50^, it appears unlikely that EV can effectively be filtered by any dialysis procedure. It has been observed that MP counts rise after one hour of dialysis^51^ most likely as a sign of bioincompatibility of the procedure. However, Ruzicka et al. found reduced levels of MP after completion of a 4-h HFL dialysis session^52^. The proposed mechanism was adsorption of MP to the dialysis membrane^52^. This body of evidence raises the possibility that HCO dialysis might promote a persistent reduction in MP with pro-calcifying activity on MSC and VSMC due to dampening effects on inflammation, enhanced MP extraction or both. In addition, certain URS can stimulate the production of MP with characteristically altered compounds turning them into even more harmful vehicles with influences on remote targets such as endothelium, VSMC and coagulation^53^. HCO dialysis could advantageously modify the composition of those MP because this modality can remove several URS with higher efficiency.

In summary, we clearly demonstrated a substantial change in functional properties of plasma from patients undergoing HCO dialysis compared to HFL dialysis resulting in profoundly reduced pro-calcifying activity on MSC. Several pro-inflammatory mediators and LMM with the potential to stimulate osteoblastic differentiation of MSC such as FGF23 were removed more effectively by HCO dialysis and we found evidence of persistently reduced inflammation. Although we were not able to specify the agents that conferred protection or whose enhanced reduction caused less harm, we propose a model where a greater transient relief from uremic toxicity in general and pro-inflammatory stimuli in particular initiates long-standing beneficial mechanisms that help preserve the regenerative potential of MSC and prevent vascular calcification. These mechanisms might include a favorable impact on MP counts and their composition. Clinical application of HCO dialysis is limited by significant albumin losses, but recently developed MCO membranes used in expanded hemodialysis provide a similar clearance of LMM with an acceptable loss of albumin^54^. However, whether or not these promising new dialysis techniques can substantially reduce cardiovascular damage and significantly improve patient outcome needs to be addressed by subsequent clinical studies.

## Methods

All studies involving human material were conducted in accordance with the Declaration of Helsinki and had been approved by local ethic authorities. The clinical trial involving dialysis patients was approved by the ethics committee of the medical faculty of the Martin-Luther-Universität Halle-Wittenberg (#2011-78). Isolation of MSC from bone marrow donors was approved by the ethics committee of the Ärztekammer Hamburg (#2572). All subjects provided written informed consent.

### Clinical trial comparing HCO and HFL dialysis membranes

We conducted a sub-study on patients from a randomized open-label clinical trial involving 43 stable chronic dialysis patients that has been described previously in detail^6^. Briefly, patients were dialyzed in a crossover design for 3 weeks each with conventional HFL dialyzers (Polyflux 210H, Gambro) and with HCO dialyzers (HCO 1100, Gambro) characterized by a higher molecular weight cut-off (experimental phases 1 and 2). Experimental phases were separated by a wash out-phase of 2 weeks with conventional HFL dialysis. The 19 patients included in this sub-study had a median age of 73 (range 50–84) years. 11 (57.9%) were male, 8 (42.1%) were female. Baseline blood samples were drawn at the first dialysis session of each experimental phase pre- and post-dialysis. Another sample was obtained pre-dialysis at the end of each 3-week experimental phase.

### Analysis of blood samples

Routine parameters were analyzed in the central clinical chemistry laboratory of the University Hospital of the Martin-Luther-University Halle. Inflammation related proteins were quantified as described in the original publication of the trial^6^.

Isolation and culture of MSC, induction of osteoblastic differentiation, measurement of ALP activity and calcium deposition, alizarin staining, immunocytochemistry and Western Blot analyses were performed following the same protocols used in previous studies^17,35^.

### Isolation and culture of MSC

MSC can be isolated from all vascularized tissues^12^ where they reside as pericytes in the vessel wall and function as vascular progenitor cells^55^. Bone marrow is an easily accessible and well established source providing sufficient numbers of cells. Bone marrow aspirates were acquired from 20 healthy donors of allogenic bone marrow transplants (7 female, 13 male) with a median age of 31 (range 0.5–42) years. MSC were isolated as described previously^56^. In brief, bone marrow mononuclear cells were purified by Ficoll density gradient centrifugation, plated at 400,000 cells/cm2 and cultured in α-MEM (#E15-862, PAA) supplemented with 100 U/mL penicillin (PAA), 100 μg/mL streptomycin (PAA), 2 IU/ml heparin (Ratiopharm), and 5% freshly thawed platelet lysate (PL) at 37 °C and 5% CO_2_. Nonadherent cells were washed off with PBS after 2–3 days. Medium was changed twice a week. When cultures reached about 70% confluence, cells were detached with 0.05% Trypsin/0.02% EDTA (PAA), counted, and re-plated at 500 cells/cm^2^ in 175 cm^2^ flasks (Sarstedt). All MSC preparations were tested for expression of a characteristic surface marker profile (positive for CD73, CD90, and CD105; negative for CD11b, CD14, CD19, CD34, CD45, and HLA-DR). Multilineage differentiation capacity into adipocytes, chondrocytes, and osteoblasts was confirmed according to the standard criteria for the definition of MSC^57^.

### Induction of osteoblastic differentiation

MSC (passages 2 to 5) were seeded in complete α-MEM at 140,000 cells per well in 6-well plates. Medium was changed the following day to osteoblast induction medium (OM) consisting of Dulbecco’s Modified Eagle’s Medium (DMEM; PAA) supplemented with 2 mM glutamine (PAA), penicillin/streptomycin (PAA), 1% FCS (PAA), 10 mM β-glycerophosphate (Applichem), 500 µM ascorbic acid, and 100 nM dexamethasone (all from Sigma). Medium was subsequently changed every 2–3 days.

In the experiments with patient plasma, FCS in OM was replaced by 2.5% patient plasma throughout the whole experiment with medium changes every 2–3 days. Plasma was extracted from whole blood by centrifugation and was used without any further processing.

### Alkaline phosphatase activity

Activity of ALP in MSC was measured after exposure to the different experimental conditions for 7 days. After washing with PBS, cells were lysed with 400 µl ALP lysis buffer (150 mM Tris pH 10.0, 0.1 mM ZnCl2, 0.1 mM MgCl2, 1% Triton-X100) at room temperature under constant agitation for 30 min. Supernatants were immediately frozen at − 80 °C. For measurement of ALP activity, an aliquot was centrifuged for 10 min at 12,000 rpm and 4 °C. Each sample was measured in triplicate. 50 µl per well were mixed with 200 µl pre-warmed (37 °C) substrate solution (ALP buffer with freshly dissolved p-Nitrophenyl phosphate at 2.7 mM) in 96-well plates. Optical densities (OD) were measured at 405 nm and followed over a 60 min incubation period at 37 °C in intervals of 10 min. A time point during the linear phase was chosen and ∆OD values to baseline ODs were calculated and divided by the protein concentration of the sample as determined with the DC Protein Assay (Bio-Rad) for normalization. Finally, each ∆OD/protein ratio was related to the ∆OD/protein ratio of the appropriate control.

### Calcium deposition

Deposition of extracellular calcium by MSC undergoing osteoblastic differentiation was assessed after 3 weeks of incubation with OM supplemented with the indicated experimental constituents. Calcified cells were scraped off in 500 µL 0.6 M HCl and incubated in microtubes overnight under constant agitation at 4 °C to solubilize the calcium. Samples were centrifuged for 60 min at 20,000*g* and 4 °C. Supernatants were transferred to new microtubes for calcium measurement. Pellets were dissolved in 25 µl 0.1 M NaOH/0.1% SDS solution for protein quantification with the DC protein assay (Bio-Rad). Supernatants were analyzed in duplicate in 96-well plates and compared to a calcium standard curve. 10 µL were mixed with 150 µL color reagent (0.1 mg/mL ortho-cresophthalein complexone, 1 mg/mL 8-hydroxy-quinoline, 0.7 M HCl) and 150 µl AMP buffer (15% 2-amino-2-methyl-1-propanol in H2O, pH 10.7) and incubated for 15 min at room temperature. ODs were measured at 540 nm. Calcium concentrations were calculated by means of the standard curve and normalized to protein content.

### Alizarin staining

140,000 MSC per well were seeded in 6-well plates in 1% FCS/DMEM and allowed to adhere overnight. After incubation for 3 weeks as indicated, cells were fixed with ice-cold methanol for 30 min at − 20 °C and air dried. 5% Alizarin (1,2-dihydroxyanthraquinone, Sigma) in 0.1 M boric acid buffer (pH 4) was filtered and applied to the cells for 1 h at room temperature. After several wash steps with PBS (pH 6.0), wells were dried and micrographs were taken on a Zeiss Axiovert 40 CFL using a Canon PowerShot A649.

### Immunocytochemistry

50,000 cells per well were seeded on 15 mm glass-coverslips (Roth) placed in 12-well plates in 1% FCS/DMEM and allowed to adhere overnight. After incubation for 3 weeks as indicated, cells were fixed with 4% paraformaldehyde (Sigma) in PBS for 10 min, and permeabilized for 3–5 min with 0.5% Triton X-100 (Applichem) in PBS. After blocking overnight in 3% BSA/PBS at 4 °C, primary antibodies (Osteopontin abcam ab8448, Collagen I abcam ab34710; 1:500 in blocking solution) were incubated for 2 h at 37 °C in a wet chamber. After three washes with PBS, slides were incubated with appropriate secondary antibodies (HRPO-conjugated IgG, Dianova) for 2 h at room temperature. Signal was developed with AEC High Sensitivity Substrate Chromogen Ready to use (Dako) for about 10 min. Coverslips were washed extensively, and cells were counterstained with Mayer’s hematoxylin (Medite) followed by a final wash in water pH 12.6 with NaOH. Photomicrographs were taken as described above.

### Western blot

360,000 cells were incubated in 6 cm dishes for 3 weeks as indicated. Cells were lysed (20 mM Tris pH 7.5, 350 mM NaCl, 1% Triton X-100, 1 × Roche complete protease inhibitor cocktail, 1 mM PMSF, 1 mM sodium orthovanadate, 10 nM β-glycerophosphate, 5 mM NaF) for 20 min on ice. After centrifugation, protein concentrations in the supernatants were quantified with the DC protein assay (Bio-Rad). 5 × Laemmli buffer (250 mM Tris pH 6.8, 500 mM DTT, 10% SDS, 0.5% Bromophenol blue, 35% Glycerol) was added, and samples were heated to 99 °C for 5 min. 50 µg total protein per lane were separated by SDS-polyacrylamid-gel-electrophoresis and electrotransferred to a PVDF membrane (GE Healthcare) following standard protocols. Blocking was performed with 10% BSA /0.1% Triton X-100/TBS for 2 h at room temperature. All antibodies were diluted in blocking solution: Collagen I (abcam) and Cbfa/Runx (MBL) 1:500; Osteopontin (abcam) 1:1000. GAPDH (hytest) served as a loading control and was applied at 1:100,000. After incubation with appropriate secondary antibodies (Dianova), SuperSignal West Pico Chemiluminescent Substrate (Thermo Fisher) was used for development in a G:BOX F3 device (Syngene).

### Panel of individual URS

To screen for biologic effects of URS on MSC, individual URS were tested at the highest concentrations reported in patients with chronic renal failure requiring renal replacement therapy (c_max_) as suggested in the 2003 and 2007 EUTox reports^23,58^ and subsequent publications on uremic toxicity^25,59,60^. Appropriate solvent controls were included in all experiments. Protein bound URS were applied in presence of 35 g/L human albumin as recommended by EUTox^23^. For details see Table S1.

### Cell proliferation assay

5000 cells per well were seeded in 96-well plates in complete α-MEM and allowed to adhere overnight. The next day, medium was changed to α-MEM without FCS or PL. 24 h later, cells were exposed to the URS at the concentrations listed in Table S1 in α-MEM without FCS or PL in presence of BrdU (1:500). BrdU incorporation as a surrogate for proliferation was measured after incubation for another 24 h with the BrdU cell proliferation kit (Roche) following the manufacturer’s instructions. Each independent experiment consisted of five replicates for every measurement. Results were expressed relative to the appropriate solvent control.

### MTT

3-(4,5-Dimethyl-2-thiazolyl)-2,5-diphenyl-2H-tetrazoliumbromid (MTT) was dissolved to a concentration of 5 g/L in 0.9% NaCl solution and sterile filtered with 0.22 µm filters. 24 h after seeding of 4500 cells per well in 96-well plates in complete α-MEM, incubation with URS at the concentrations listed in Table S1 in α-MEM without FCS or PL was started. 0.1% sodium azide was used a positive control. After 24 h, 10 µl MTT solution per well were added and incubation was continued for 4 h. Wells were washed with PBS and formazan crystals formed by metabolically active cells were dissolved in 100 µl isopropanol. Absorbance was measured in a microplate reader at a wavelength of 590 nm and 690 nm as a reference. After subtraction of background absorbance, means of five replicates were calculated and expressed relative to the appropriate solvent control.

### Statistics

All data are detailed as median with 25% and 75% quartiles in brackets unless otherwise indicated. The Wilcoxon matched pairs test was applied to compare the effects of plasma obtained after HFL and HCO dialysis on osteoblastic differentiation of MSC and to test for differences in reduction rates of HFL and HCO membranes. Statistical comparison of multiple groups was performed with the Kruskal–Wallis test or the Friedman test when a paired analysis was appropriate. Dunn’s Multiple Comparisons Test was used as a post hoc test. 1-way ANOVA followed by Dunnett’s post hoc test was used to evaluate dose–response curves. Significance was considered at a value of *P* < 0.05. All analyses were performed with GraphPad Prism version 5.02 for Windows, GraphPad Software, San Diego California USA.

## Supplementary Information

Supplementary Figures.

Supplementary Information.
