# Supplementary material for: High cut-off dialysis mitigates pro-calcific effects of plasma on vascular progenitor cells
Source: Sci Rep. 2021 Jan 13;11:1144. doi: 10.1038/s41598-020-80016-7 (PMC7807056; doi:10.1038/s41598-020-80016-7)
Supplement: Supplementary file 1 — Supplementary Figures. [file 41598_2020_80016_MOESM1_ESM.docx]

**High cut-off dialysis mitigates pro-calcific effects of plasma on vascular progenitor cells**

Theres Schaub^1,2^, Daniel Janke^1^, Daniel Zickler^1^, Claudia Lange^3^, Matthias Girndt^4^, Ralf Schindler^1^, Duska Dragun^1,5,6^, Björn Hegner^1,5,6,7^

^1^Charité – Universitätsmedizin Berlin, corporate member of Freie Universität Berlin, Humboldt-Universität zu Berlin, and Berlin Institute of Health, Clinic for Nephrology and Intensive Care Medicine, Campus Virchow-Clinic, Berlin, Germany

^2^Charité – Universitätsmedizin Berlin, corporate member of Freie Universität Berlin, Humboldt-Universität zu Berlin, and Berlin Institute of Health, Institute of Cell Biology and Neurobiology, Berlin, Germany

^3^Clinic for Stem Cell Transplantation, Department of Cell and Gene Therapy, University Medical Center Hamburg-Eppendorf, Hamburg, Germany

^4^Department of Internal Medicine II, Martin-Luther-University Halle-Wittenberg, Halle, Germany

^5^Berlin-Brandenburg School for Regenerative Therapies (BSRT), Berlin, Germany

^6^Center for Cardiovascular Research (CCR), Charité University Hospital, Berlin, Germany

^7^Vivantes Ida Wolff Hospital for Geriatric Medicine, Berlin, Germany

Supplementary information

Full length blots


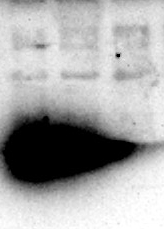

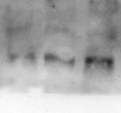


osterix

Cbfa


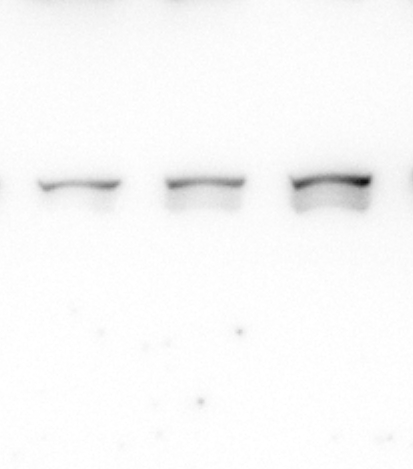

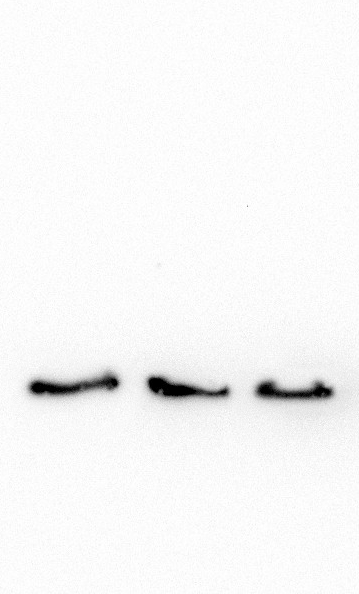

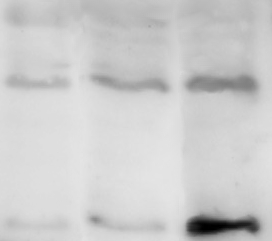


GAPDH

Osteopontin

Collagen
