# Supplementary material for: High cut-off dialysis mitigates pro-calcific effects of plasma on vascular progenitor cells
Source: Sci Rep. 2021 Jan 13;11:1144. doi: 10.1038/s41598-020-80016-7 (PMC7807056; doi:10.1038/s41598-020-80016-7)
Supplement: Supplementary file 2 — Supplementary Information. [file 41598_2020_80016_MOESM2_ESM.docx]

**High cut-off dialysis mitigates pro-calcific effects of plasma on vascular progenitor cells**

Theres Schaub^1,2^, Daniel Janke^1^, Daniel Zickler^1^, Claudia Lange^3^, Matthias Girndt^4^, Ralf Schindler^1^, Duska Dragun^1,5,6^, Björn Hegner^1,5,6,7^

^1^Charité – Universitätsmedizin Berlin, corporate member of Freie Universität Berlin, Humboldt-Universität zu Berlin, and Berlin Institute of Health, Clinic for Nephrology and Intensive Care Medicine, Campus Virchow-Clinic, Berlin, Germany

^2^Charité – Universitätsmedizin Berlin, corporate member of Freie Universität Berlin, Humboldt-Universität zu Berlin, and Berlin Institute of Health, Institute of Cell Biology and Neurobiology, Berlin, Germany

^3^Clinic for Stem Cell Transplantation, Department of Cell and Gene Therapy, University Medical Center Hamburg-Eppendorf, Hamburg, Germany

^4^Department of Internal Medicine II, Martin-Luther-University Halle-Wittenberg, Halle, Germany

^5^Berlin-Brandenburg School for Regenerative Therapies (BSRT), Berlin, Germany

^6^Center for Cardiovascular Research (CCR), Charité University Hospital, Berlin, Germany

^7^Vivantes Ida Wolff Hospital for Geriatric Medicine, Berlin, Germany

Supplementary information

| **Solute** | **c_max_ [/L]** | | **Solvent** | **Supplier** | **Reference** |
| --- | --- | --- | --- | --- | --- |
| 1-Methyladenosine | 216.4 | µg | 0.9% NaCl | Sigma-Aldrich | [46](#_ENREF_47) |
| α-N-Acetylarginine | 4.58 | mg | 0.9% NaCl | Sigma-Aldrich | [46](#_ENREF_47) |
| Arabitol | 33 | mg | 0.9% NaCl | Sigma-Aldrich | [46](#_ENREF_47) |
| Benzylalcohol | 187.9 | mg | 0.9% NaCl | Sigma-Aldrich | [46](#_ENREF_47) |
| Connective Tissue Growth Factor | 100 | µg | NaAcetat | Immunotools | [47](#_ENREF_48) |
| Creatinine | 240 | mg | 0.9% NaCl | Merck-Millipore | [46](#_ENREF_47) |
| Dimethylglycine | 1.04 | mg | 0.9% NaCl | Sigma-Aldrich | [46](#_ENREF_47) |
| Endothelin-1 | 129.4 | ng | ddH_2_O | Sigma-Aldrich | [46](#_ENREF_47) |
| Erythritol | 34 | mg | 0.9% NaCl | Sigma-Aldrich | [46](#_ENREF_47) |
| Fibroblast Growth Factor 23 | 255.2 | ng | 0.1% BSA/ddH_2_O | R&D | [25](#_ENREF_26) |
| γ-Guanidinobutyric acid | 1.75 | mg | 0.9% NaCl | Sigma-Aldrich | [46](#_ENREF_47) |
| Guanidine.HCl | 1.29 | mg | 0.9% NaCl | Sigma-Aldrich | [46](#_ENREF_47) |
| Guanidinoacetic acid | 694 | µg | 0.9% NaCl | Sigma-Aldrich | [46](#_ENREF_47) |
| Hippuric acid | 471 | mg | 0.25 M NaOH/Tris | Sigma-Aldrich | [46](#_ENREF_47) |
| Hydroquinone | 286 | µg | 0.9% NaCl | Sigma-Aldrich | [46](#_ENREF_47) |
| Hypoxanthine | 5.3 | mg | 0.25 M NaOH/Tris | Sigma-Aldrich | [46](#_ENREF_47) |
| Indole-3-acetic acid | 9.08 | mg | 20% Ethanol | Sigma-Aldrich | [46](#_ENREF_47) |
| Indoxyl sulfate potassium salt | 279.5 | mg | 0.9% NaCl | Sigma-Aldrich | [46](#_ENREF_47) |
| Interleukin-1β | 1.7 | µg | 0.1% BSA/ddH_2_O | Immunotools | [46](#_ENREF_47) |
| Interleukin-6 | 328.1 | ng | 0.1% BSA/ddH_2_O | Immunotools | [46](#_ENREF_47) |
| Kynurenic acid | 9.5 | mg | 0.1 M NaOH | Sigma-Aldrich | [46](#_ENREF_47) |
| Mannitol | 76 | mg | 0.9% NaCl | Sigma-Aldrich | [46](#_ENREF_47) |
| Myoinositol | 232 | mg | 0.9% NaCl | Sigma-Aldrich | [46](#_ENREF_47) |
| p-OH-Hippuric acid | 31.5 | mg | MeOH | Bachem | [46](#_ENREF_47) |
| Tumor Necrosis Factor-α | 408 | ng | 0.1% BSA/ddH_2_O | Immunotools | [46](#_ENREF_47) |
| Urea | 4.6 | g | 0.9% NaCl | Sigma-Aldrich | [46](#_ENREF_47) |
| Uric acid | 146.7 | mg | 0.25 M NaOH/Tris | Sigma-Aldrich | [46](#_ENREF_47) |
| Uridine | 32.6 | mg | 0.2 M NaOH/Tris | Sigma-Aldrich | [46](#_ENREF_47) |
| Xanthosine | 222.4 | µg | 0.1 M NaOH | Sigma-Aldrich | [46](#_ENREF_47) |

Supplementary Table 1

25. Duranton, F, Cohen, G, De Smet, R, Rodriguez, M, Jankowski, J, Vanholder, R, Argiles, A: Normal and pathologic concentrations of uremic toxins. *J Am Soc Nephrol,* 23**:** 1258-1270, 2012.

46. Vanholder, R, Meert, N, Schepers, E, Glorieux, G, Argiles, A, Brunet, P, Cohen, G, Drueke, T, Mischak, H, Spasovski, G, Massy, Z, Jankowski, J: Review on uraemic solutes II--variability in reported concentrations: causes and consequences. *Nephrol Dial Transplant,* 22**:** 3115-3121, 2007.

47. Gerritsen, KG, Abrahams, AC, Peters, HP, Nguyen, TQ, Koeners, MP, den Hoedt, CH, Dendooven, A, van den Dorpel, MA, Blankestijn, PJ, Wetzels, JF, Joles, JA, Goldschmeding, R, Kok, RJ: Effect of GFR on plasma N-terminal connective tissue growth factor (CTGF) concentrations. *Am J Kidney Dis,* 59**:** 619-627, 2012.
